# Guanidyl-Rich Poly(β Amino Ester)s for Universal Functional Cytosolic Protein Delivery and Clustered Regularly Interspaced Short Palindromic Repeats (CRISPR) Cas9 Ribonucleoprotein Based Gene Editing

**DOI:** 10.1021/acsnano.3c03269

**Published:** 2023-09-05

**Authors:** Xianqing Wang, Yinghao Li, Xi Wang, Dario M. Sandoval, Zhonglei He, Sigen A, Irene Lara Sáez, Wenxin Wang

**Affiliations:** † Charles Institute of Dermatology, School of Medicine, University College Dublin, D04 V1W8 Dublin, Ireland; ‡ Research and Clinical Translation Center of Gene Medicine and Tissue Engineering, School of Public Health, Anhui University of Science and Technology, Huainan 232001, China

**Keywords:** cytosolic protein delivery, poly(β amino ester)s, guanidyl, CRISPR/Cas9 ribonucleoprotein transfection, gene editing

## Abstract

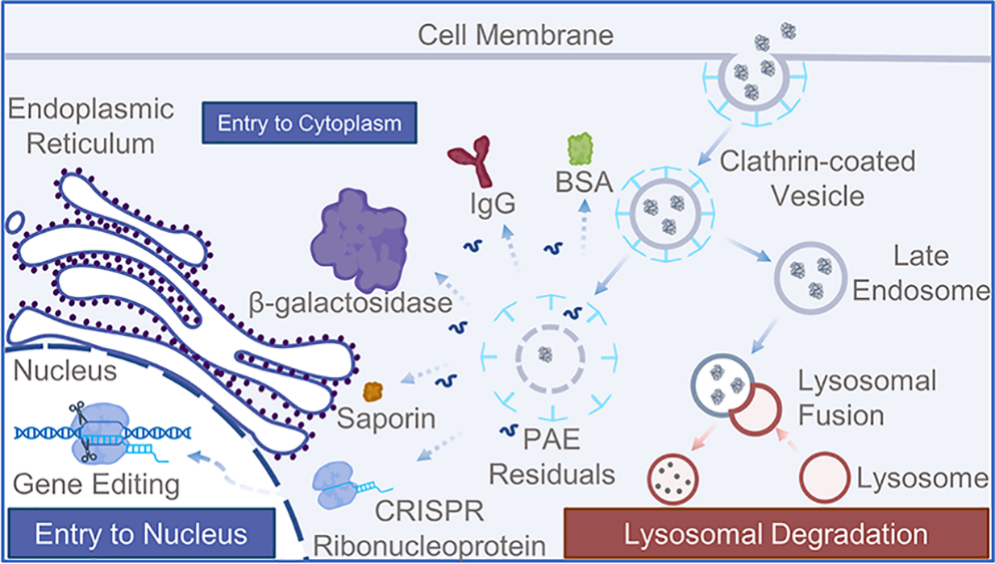

Protein therapeutics are highly promising for complex disease treatment. However, the lack of ideal delivery vectors impedes their clinical use, especially the carriers for *in vivo* delivery of functional cytosolic protein. In this study, we modified poly(β amino ester)s (PAEs) with a phenyl guanidine (PG) group to enhance their suitability for cytosolic protein delivery. The effects of the PG group on protein binding, cell internalization, protein function protection, and endo/lysosomal escape were systematically evaluated. Compared to the unmodified PAEs (L3), guanidyl rich PAEs (L3PG) presented superior efficiency of protein binding and protein internalization, mainly via clathrin-mediated endocytosis. In addition, both PAEs showed robust capabilities to deliver cytosolic proteins with different molecular weight (ranging from 30 to 464 kDa) and isoelectric points (ranging from 4.3 to 9), which were significantly improved in comparison with the commercial reagents of PULsin and Pierce Protein Transection Reagent. Moreover, L3PG successfully delivered Clustered Regularly Interspaced Short Palindromic Repeats (CRISPR) Cas9 ribonucleoprotein (RNP) into HeLa cells expressing green fluorescent protein (GFP) and achieved more than 80% GFP expression knockout. These results demonstrated that guanidyl modification on PAEs can enhance its capabilities for intracellular delivery of cytosolic functional proteins and CRISPR/Cas9 ribonucleoprotein. The guanidyl-rich PAEs are promising nonviral vectors for functional protein delivery and potential use in protein and nuclease-based gene editing therapies.

## Introduction

Protein therapeutics have overwhelming advantages over small molecule drugs in many aspects: (1) highly particular and complex functions that cannot be mimicked by simple chemical compounds, (2) very specific in biological processes with less adverse effects, and (3) naturally occurring in the body to reduce the potential of immune response.^1^ Therefore, protein therapeutics are highly promising for various disease treatments by either effective replacement of the key protein,^2−4^ or gene editing using nuclease-guided machinery such as Zinc Finger Nuclease (ZFNs),^5,6^ Transcription Activator-Like Effector Nucleases (TALEN),^7,8^ and Clustered Regularly Interspaced Short Palindromic Repeats-Associated (CRISPR/Cas) Nucleases.^9,10^

However, the clinical use of protein therapeutics, in particular, nuclease-based gene editing therapeutics, is still rare due to the tough challenge of seeking ideal techniques for protein delivery. Intracellular protein delivery is even more difficult than protein delivery for extracellular targets, as it requires crossing the cell membrane, entering the cytosol, and accessing targeted organelles such as the nucleus. The cell membrane is the first barrier that is impermeable to most proteins. Although carriers such as liposomes,^11−13^ polymers,^14−17^ inorganic nanoparticles,^18,19^ and exosomes^20,21^ have been developed to transport protein cargos into the cytosol mainly via endocytosis, and the endocytosis could further lead to endo/lysosome entrapment, which is the secondary barrier and seriously impedes intracellular protein transportation.^22,23^ Only few cargos or carriers can escape from endo/lysosomes while most of them often are degraded or exocytosed.^24^ Furthermore, the dynamic functional protein concentration in the cytosol means cytosolic protein-based therapies rarely achieve high efficacy, since following escape from endo/lysosomes, target sites need to be accessed before degradation in the cytosol.

To overcome those barriers and facilitate intracellular protein delivery, two main strategies have been adopted. One of the strategies is to modify proteins with membrane permeable species such as membrane penetrant peptides, signaling ligands, and polymers to increase their interaction with membranes and eventually penetrate the cell membrane and endosome.^25,26^ Alternatively, functional modification is introduced to carriers, which benefits transmembrane transportation and cargo protection during the endocytosis. Multiple research groups have reported modification strategies of carriers to efficiently transport proteins into the cytosol, including guanidinium-rich,^27−31^ fluorinated,^32−34^ boronic acid-rich,^14,29^ and amphiphilic^15^ engineering. Guanidine groups have potential for translocating numerous biologically active substances across cellular membranes, as they are widely found on cell-penetrating peptides, protein transduction domains, and numerous synthetic mimics.^35^ Grafting guanidyl ligands onto nanoparticles provides a multivalent effect on protein binding through electrostatic forces, salt bridge, and hydrogen bonding interactions. Lv et al.^29^ examined a library of guanidyl-rich polyethylenimine (PEI) with different kinds of hydrophobic ligands and found that 4-guanidinobenzoic acid (GBA) is critical for stabilization of the polymer/protein complexes. More recently, Barrios et al.^36^ reported that a direct conjugation of planar carbamoyl group to guanidine can decrease acid dissociation constant (p*K*_a_) of the polymer, increase local hydrophobicity and hydrogen bonding interactions, and overall eventually enhance the protein complex stability in serum-containing media.

Poly(β amino ester)s (PAEs) was reported as gene delivery vector by Lynn and Langer et al. in 2000.^37^ Since then, both linear and highly branched PAEs have been widely used for nucleic acid delivery,^38−42^ but they are rarely reported as protein delivery vectors. PAEs and other traditional cationic polymers self-assemble nucleic acids mainly through electrostatic interactions, which are generally recognized as insufficient to encapsulate proteins of diverse surface charge. To overcome this limitation of PAEs, phenylboronic acid, carboxylate ligands, and zwitterionic phosphocholine have been introduced to PAEs to obtain varied interactions between the polymer and protein.^15,29,43,44^ In this study, we demonstrated that the phenyl guanidine (PG) group end-capped PAEs (L3PG) can enhance polymer–protein interaction, increase complex stability, and improve intracellular protein delivery efficiency. We, therefore, systematically evaluated the effect of guanidyl-rich PAEs on protein binding, intracellular protein delivery, endocytosis pathway, endo/lysosomal entrapment, and protein function protection. On the delivery of protein ranging from 30 kDa to 464 kDa in molecular weight with varying surface charge (isoelectric points (pI) from 4.3 to 9), the guanidyl-rich PAEs showed robust protein delivery ability, superior to the control PAEs and commercial reagents like PULsin and Pierce Protein Transection Reagent (Pierce). The guanidyl-rich PAEs also demonstrate an exceptional capacity for CRISPR/Cas9 ribonucleoprotein (RNP) delivery for efficient gene editing. The PG modification contributed to more than 80% down-regulation of green fluorescent protein (GFP) expression in GFP positive HeLa cells (HeLa-GFP).

## Results and Discussion

### Guanidyl-Rich PAEs in Protein Delivery

**Figure 1:**
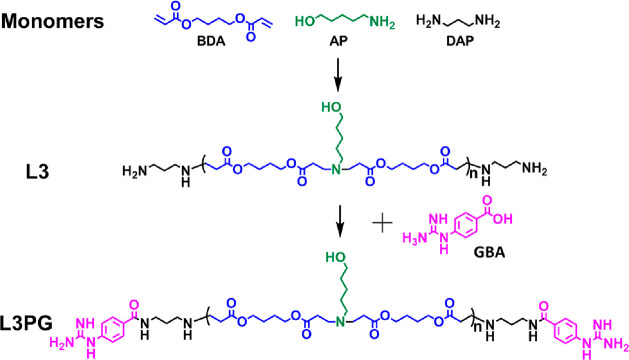
Structure of monomers and PAEs (L3 and L3PG) used in this study.

A linear PAEs, L3, was synthesized via Michael addition polymerization using a well-optimized monomer combination and targeting the ideal molecular weight for protein delivery (data not shown here). L3PG was synthesized from L3 by further reaction with GBA as a cationic terminal group (Figure 1). The successful GBA modification was confirmed by the presence of phenyl (6.28 and 6.96 ppm) and guanidine (5.42, 7.18, and 7.81 ppm) protons in ^1^H NMR (Figures S1 and S2 in the Supporting Information). PG ligands, capable of forming electrostatic interactions, hydrogen bonds, and salt bridges with proteins, were predicted to enhance the protein delivery capacity of the modified PAEs.

**Figure 2:**
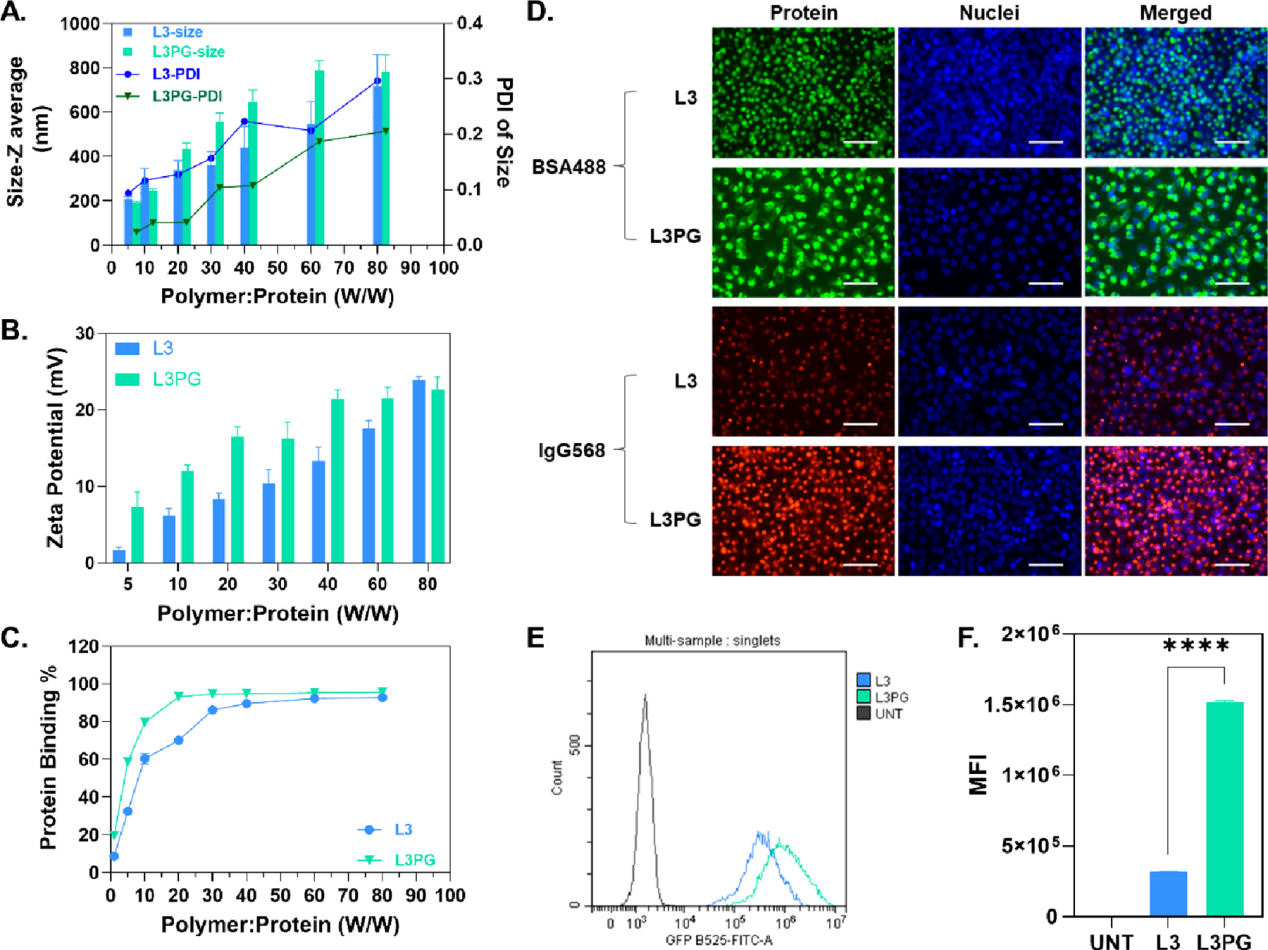
PAEs and protein complexes characterization and cellular uptake. (A) Size of PAEs/BSA complexes. (B) Zeta potential of PAEs/BSA complexes. (C) Protein binding efficiency of PAEs. (D) Representative fluorescence images of Hela cells transfected by PAEs/BSA-FITC and PAEs/IgG-FITC complexes (W/W of 20 and 30 for L3PG and L3, respectively). BSA-FITC was indicated in green, IgG-FITC was shown in red, and nuclei were stained by Hoechst 33342 and shown in blue. Scale bar = 400 μm. (E) Fluorescence histogram of flow cytometry data from HeLa cells transfected with PAEs/BSA-FITC complexes (W/W = 20 and 30 for L3PG and L3, respectively). (F) MFI of HeLa cells treated with PAEs/BSA-FITC complexes (W/W = 20 and 30 for L3PG and L3, respectively). Data represented as mean ± SD (*n* = 3), (****) *P* < 0.0001, one-way analysis of variance (ANOVA) with Tukey’s multiple comparison test.

To investigate the protein encapsulation capabilities of PAEs, bovine serum albumin (BSA, 66 kDa, pI 4.6) was selected as a model protein to form PAEs/protein complexes. At PAEs to protein weight ratios (W/W) ranging between 5 and 80, both L3PG and L3 could assemble with BSA into nanoparticles, the hydrodynamic diameter of which ranged from 200 to 800 nm with positive surface charge (see Figures 2A and 2B). Furthermore, the nanoparticle size was increased as the W/W ratio increased, and the L3PG/BSA complex generally had slightly bigger size than L3/BSA. Transmission electron microscopy (TEM) images of the complexes at W/W = 20 also confirmed complex formation (Figure S3 in the Supporting Information). It was notable in the TEM images that nanoparticles ∼200 nm in diameter were an aggregation of numerous smaller polyplexes, suggesting that the smaller polyplexes are formed at the beginning and then tend to accumulate together toward forming more stable and bigger nanoparticles. This could be explained by the balance of the interparticle hydrophobic interaction and ionic repulsion. Along with the increased polymer-to-protein ratio, the higher surface positive charge increases the interparticle repulsion. However, the strong hydrophobic interaction enforces the formation of aggregation, therefore increasing the size of complexes. The comparison revealed that the PG modification, which promotes the hydrophobic interaction of polymers and hydrogen-bond interaction between proteins and PAEs,^11,45^ could results in more compact nanoparticles with narrower polydispersity index (PDI), thus improves the complex stability. The protein encapsulation capabilities were quantified with fluorescein isothiocyanate (FITC)-labeled BSA (BSA-FITC). As expected, L3PG showed superior protein binding ability, of which ∼95% of proteins were encapsulated at W/W = 20. Although L3 could achieve a similar protein binding capacity (around 93%), the W/W ratio had to be increased to over 30 (Figure 2C). These data indicated the advantage of PG ligand in encapsulating proteins. The superiority of L3PG was also displayed in protein cellular internalization as it achieved five times more BSA-FITC internalization than L3 (*p* < 0.0001) (Figures 2E and 2F). BSA-FITC and IgG-FITC (160 kDa, pI 4.3) were successfully delivered by both L3PG and L3 into HeLa cells (Figure 2D). The quantitative data (Figure S4 in the Supporting Information) showed that the protein uptake amount decreased with increasing W/W ratio. It should be noted that increasing protein encapsulation (with optimal encapsulation achieved at W/W = 20 and 30 for L3PG and L3, respectively) did not result in a corresponding increase of protein uptake level. On the other hand, the high level of PAEs/protein complex uptake did not lead to obvious cytotoxicity, since the viability of treated cells was over 80% for both PAEs, L3 and L3PG, at the W/W below 30 (PAEs concentration <0.12 mg/mL) (Figure S5 in the Supporting Information).

### Internalization Pathway of PAEs/Protein Complexes

**Figure 3:**
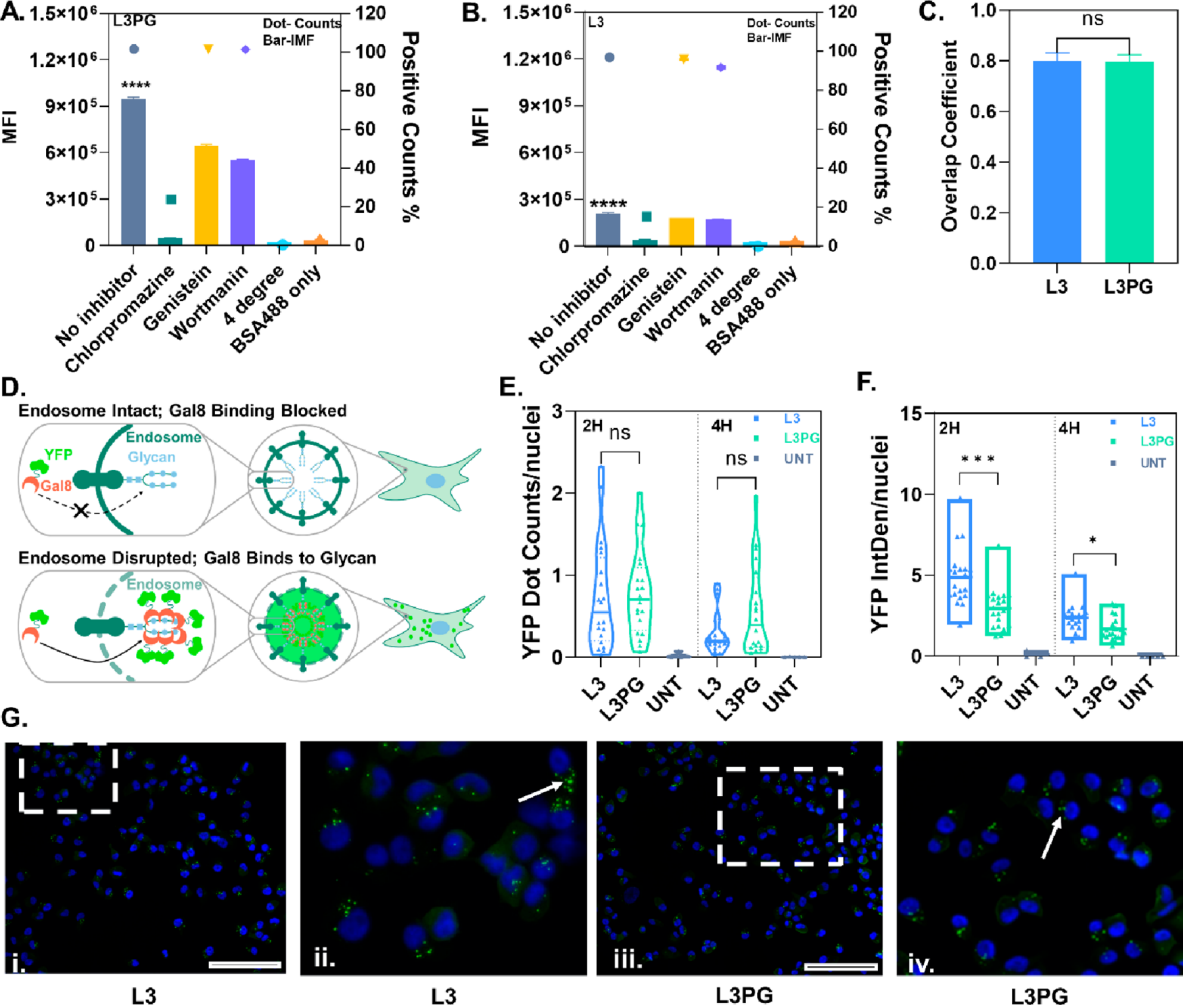
Endocytosis pathway and endo/lysosomal escape behaviors of PAEs/protein in HeLa cells. MFI of HeLa cells pretreated with endocytosis inhibitors and transfected by (A) L3PG/BSA-FITC and (B) L3/BSA-FITC. Data represent mean ± SD (*n* = 3), (****) *p* < 0.0001, one-way ANOVA with Dunnett’s multiple comparison. (C) Overlap coefficient of BSA-FITC and LysoTracker in HeLa cells at 4 h after transfection. (D) Schematic illustration of endosome disruption in HEK-Gal8YFP cells. [Adapted with permission from ref (47). Copyright 2019, American Chemical Society, Washington, DC.] (E) Fluorescent dot counts in the HEK-Gal8YFP cells 2 and 4 h after transfection, normalized by nuclei number. Data represented as mean ± SD (*n* = 3), ns, no statistical significance, one-way ANOVA with Tukey’s multiple comparison test. (F) Florescence intensity 2 or 4 h after transfection, normalized by nuclei number. Data represented as mean ± SD (*n* = 3); (*) *p* < 0.05, (***) *p* < 0.001, one-way ANOVA with Tukey’s multiple comparison test. (G) Representative fluorescent microscope images of HEK-Gal8YFP cells 2 h post-transfection: (i) HEK-Gal8YFP cells treated by L3/BSA; (ii) magnification of the area in the white rectangle on the image in panel (i), the white arrow indicated disrupted endosomes with robust fluorescence intensity; (iii) HEK-Gal8YFP cells treated by L3PG/BSA; (iv) magnification of the area in the white rectangle on the image in panel (iii); the white arrow indicated disrupted endosomes with weak fluorescence intensity. Nuclei are stained by Hoechst 33342 and shown in blue, and the disrupted endosomes are indicated in green. Scale bar = 200 μm. W/W values of 20 and 30 were used for L3PG and L3, respectively.

Although polymeric carriers are widely recognized to enter the cytosol via endocytosis, recent studies^25,28^ have proposed another theory in which the guanidyl-functionalized carriers could penetrate through the cell membrane into the cytosol. Therefore, in order to determine the internalization pathway of PAEs/protein complexes and investigate the effect of the PG group on cellular internalization, different endocytic and membrane fusion inhibitors were selected to prevent endocytosis. Flow cytometry analysis revealed that pretreatment with chlorpromazine, genistein, and wortmannin significantly inhibited the cellular uptake of PAEs/BSA-FITC complexes by ∼80%, 30%, and 40%, respectively. In addition, 4 °C incubation was confirmed to completely stop the cellular uptake because of the loss of membrane fluidity (Figures 3A and 3B).^46^ Those data indicated that PAEs/protein complexes were primarily internalized via clathrin-mediated endocytosis and partially transported by caveolin-dependent endocytosis and micropinocytosis, regardless of the presence or absence of PG modification.

### Endo/lysosomal Escape

Endo/lysosomal entrapment is the main obstacle for nonviral intracellular delivery, which impedes the cargo from being transported to the targeted location and thus results in an inefficient therapeutic effect. In this study, fluorescent confocal images of HeLa cells treated with PAEs/BSA-FITC for 4 h revealed diffused BSA-FITC signal throughout the cytosol with clusters of green dots (Figure S6 in the Supporting Information). The dotted fluorescence is usually considered as cargos entrapped in the endo/lysosome while the diffused fluorescence represents the free cargos in cytosol. To further evaluate the effect of PG modification on endo/lysosomal escape, the overlap coefficient of BSA-FITC and lysosome tracker were calculated using Just Another Colocalization Plugin (JACoP) in ImageJ Fiji software.^48^ The results showed that both PAEs/BSA-FITC complexes were partially entrapped in the endo/lysosomes and had similar overlap coefficients (Figure 3C).

The ability of the two PAEs/protein complexes to disrupt endosomes was also visualized on HEK-Gal8YFP cells.^47^ Galectin 8 (Gal8) is a protein dispersed in cytosol. When the endosome is disrupted, Gal8 redistributes and binds to glycosylation moieties only located on the inner membranes of endosome. By fusing fluorescent protein to Gal8, the endosomal integrity can be semiquantitatively assessed (Figure 3D). Herein, HEK-Gal8YFP cells were treated with PAEs/BSA complexes for 2 and 4 h separately, then examined by fluorescent microscope. At both time intervals, there were no significant differences of counted fluorescent dots between L3PG- and L3-treated cells (*P* > 0.05) (Figure 3E), but L3-mediated transfection had higher semiquantified fluorescent intensity than L3PG (Figure 3F). From the fluorescent images of treated HEK-Gal8YFP cells, brighter and bigger dots were found on the L3-treated cells (Figure 3G), which indicated the slightly enhanced endosomal escape ability of L3 than L3PG. It was notable that intense endosome disruption activity was observed after 2 h, suggesting that PAEs/protein complexes rapidly escape from endosomes and avoid protein degradation in endo/lysosomes.

### Intracellular Delivery of Functional Protein

**Figure 4:**
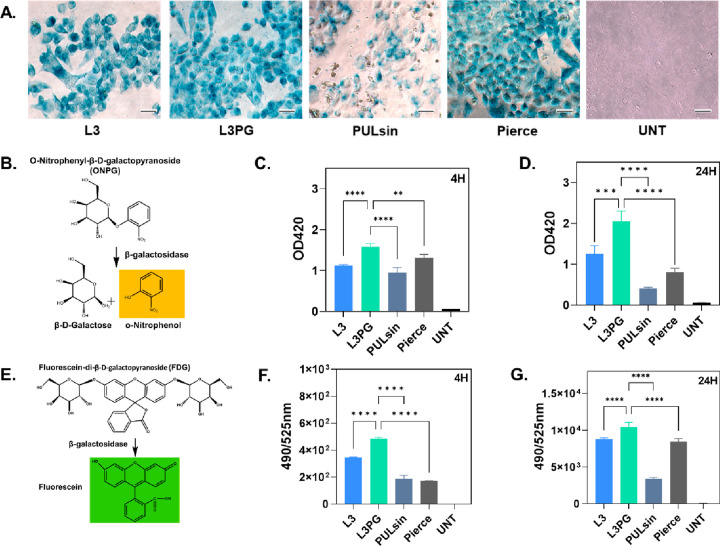
β-Gal delivery in HeLa cells. (A) Representative X-gal staining images. Scale bar = 20 μm. (B) β-Gal catalyzes the hydrolysis of colorless substrate ONPG into ONP with yellow color. (C) 4 h post-transfection, and (D) 24 h post-transfection, optical density at wavelength of 420 nm (OD420) of HeLa cells incubated with ONPG. (E) β-Gal induced cleavage of nonfluorescent FDG to produce fluorescein having spectra of *E*_x_/*E*_m_ = 490/515 nm. (F) 4 h post-transfection, and (G) 24 h post-transfection, fluorescence intensity at *E*_x_/*E*_m_ = 490/515 nm from HeLa cells incubated with FDG. Data represented as mean ± SD (*n* = 3), (**) *p* < 0.01, (***) *p* < 0.001, (****) *p* < 0.0001, one-way ANOVA with Dunnett’s multiple comparison. W/W values of 20 and 30 were used for L3PG and L3, respectively.

Unlike nucleic acids, the bioactivity of proteins may be disrupted during complexing and disassociation with polymers. The bioactivity of functional proteins is crucial for protein-based therapeutics. The protective ability of PAEs was investigated by delivering an enzymatic protein, β-galactosidase (β-Gal), in this study. β-Gal is a large negatively charged protein (464 kDa, pI 4.6), which can catalyze the hydrolysis of various β-galactosides. Commercial protein transfection reagents, PULsin and Pierce, were used as positive controls. In order to evaluate the bioactivity of β-Gal, the colorless 5-bromo-4-chloro-3-indolylb-d-galactoside (X-Gal) substrate, which could be hydrolyzed by β-Gal to generate a blue dye, was added to transfected HeLa cells. As shown in Figure 4A, cells transfected by L3PG, L3 and Pierce were stained deep blue, indicating the strong bioactivity of β-Gal. In contrast, cells treated with PULsin only had faint blue pigments, and untreated cells were not stained at all. Moreover, we also evaluated two other substrates: ortho-nitrophenyl-β-d-galactopyranoside (ONPG, can be catalyzed into ortho-nitrophenyl (ONP) anion with bright yellow color, which has a peak absorbance at 420 nm, Figure 4B) and fluorescein digalactosidease (FDG, produce a fluorescein which has a spectra of *E*_x_/*E*_m_ = 490/515 nm after catalyzation, Figure 4E) to quantify the bioactivity of β-Gal in the HeLa cells. The data showed that L3PG treated HeLa cells had superior β-Gal bioactivity (Figures 4C, 4D, 4F, and 4G) at 4 h post-transfection and even enhanced at 24 h, suggesting the great ability of PAEs to protect the protein during the endocytosis and in the cytosol.

**Figure 5:**
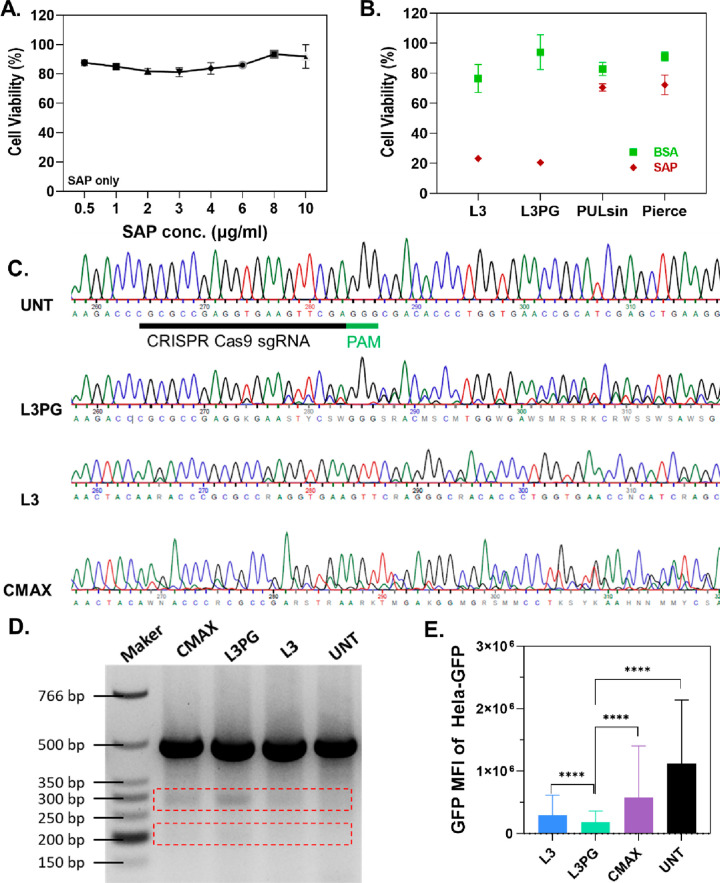
SAP and CRISPR RNP delivery. (A) Cell viability of HeLa cells treated with free SAP at concentrations from 0.5 to 10 μg/mL. (B) Cell viability of HeLa cells treated with carrier/SAP complexes and carrier/BSA complexes. (C) Sanger sequencing chromatogram of targeted GFP gene in HeLa-GFP cells. CRISPR sgRNA sequencing and protospacer adjacent motif was underlined in black and green, respectively. (D) T7E1 digestion for indels detection on the targeted gene. (E) MFI of HeLa-GFP cells treated with PAEs/RNP or CMAX/RNP at 96 h post-transfection. Data was obtained by flow cytometry. Data represented as mean ± SD (*n* = 3), (****) *p* < 0.0001, one-way ANOVA with Dunnett’s multiple comparison. W/W values of 20 and 30 were used for L3PG and L3, respectively.

Although PAEs have been proven to be able to protect the bioactivity of protein, its ability to deliver the functional protein to the targeted site and take effect still needs to be further confirmed. Therefore, saporin (SAP, 30 kDa, pI = 9) was selected as the model drug. It is a ribosome inactive enzyme that has been used for anticancer in research and clinical trials.^49^ An optimized SAP transfection condition in HeLa cells was set up with PAEs/SAP, PAEs/BSA, unloaded PAEs, and free SAP (Figure S5 in the Supporting Information). The result demonstrated that the free SAP had no cytotoxicity in HeLa cells regardless of different concentrations (Figure 5A), while L3PG/SAP and L3/SAP significantly reduced ∼80% of cell viability under their respective optimal conditions (Figure 5B). In comparison, commercial reagents/SAP showed no obvious reduction in cell viability in HeLa cells (Figure 5B). However, our developed PAEs were capable of delivering functional cytosolic protein universally to achieve its therapeutic effect, independent of the molar mass/molecule size or pI.

### Intracellular Delivery of Cas9 RNP and Gene Editing

Since the developed PAEs can deliver universal proteins robustly, we proposed that they could also be employed for CRISPR/Cas9 RNP delivery to enable gene editing. CRISPR guide RNA for GFP knock out in a GFP expressed HeLa cell line (HeLa-GFP) was designed on CRISPOR (http://crispor.tefor.net) and validated by electroporation. Sanger sequencing results showed that the guide RNA (GFP K/O-1) contributed to ∼80% of the indels at the target site (Table S3 in the Supporting Information). By transfection of the cells with complexes formed by PAEs and the CRISPR/Cas9 RNP, composed by HiFi-Cas9 nuclease and a single guide RNA (sgRNA) encoding the sequence of GFP K/O-1, L3PG/RNP achieved 19% indels at the targeted site, while the commercial CRISPR/Cas9 RNP delivery reagent, Lipofectamine CRISPRMAX (CMAX), resulted in 20% targeted indels (Figure 5C and Table S3). The targeted gene editing was also confirmed by T7 endonuclease I (T7E1) digestion (Figure 5D). Compared to L3PG, L3 only contributed to a few gene editing (6% indels), indicating that PG modification on PAEs may not only enhance the interaction between PAEs and protein but also improve the stabilization of sgRNA in the RNP complexes. CMAX achieved a higher targeted gene editing efficiency compared to L3PG and L3, whereas both PAEs resulted in greater reduction of GFP expression (Figure 5E). In contrast to the untreated HeLa-GFP cells, L3PG resulted in an 83% reduction of GFP expression, while L3 and CMAX led to reductions of 73% and 52%, respectively. Although cells treated by all the reagents had good cell viability (Figure S7 in the Supporting Information), it was observed that the transfection using PAEs resulted in cellular loss and proliferation. Therefore, the loss of GFP may occur during cell division, resulting in lower observed fluorescence intensity in the corresponding samples.^50^

## Discussion

Intracellular delivery of cytosolic functional proteins holds great value in protein therapeutics, particularly in the rapidly developed gene editing therapies using CRISPR/Cas9 nuclease. To develop a PAEs mediated protein delivery platform, we synthesized a guanidyl-rich PAEs (L3PG) using preoptimized monomer combination and a GBA ligand. As reported by other researchers, guanidinium groups could interact with carboxyl groups on proteins to form hydrogen bonds, resulting the strong combination between polymers and proteins.^30,36^ Moreover, the phenyl guanidine groups could form paired guanidinium-π interactions with each other and thus further enhance the intermolecular interaction between the polymer chains to stabilize the complexes.^30^ Those mechanisms have been demonstrated by the nanoparticles assembled by protein and L3PG, which were more compact and uniform, compared to the complexes formed with L3. The PG group end-capping on linear PAEs also resulted in higher positive surface charge on the L3PG/protein complexes, which therefore enhanced the interaction with the cell membrane. All those characteristics of L3PG significantly reflected on the protein delivery, where L3PG achieved five times higher protein cellular uptake in HeLa cells, with no effect on PAEs/protein endocytosis pathway and its endo/lysosomal escape behaviors.

Both PAEs developed here exhibited robust cytosolic protein delivery, regardless of the protein size/molar mass and pI, suggesting their potential in universal protein therapeutics. Two enzymatic proteins distinct in size and pI, β-Gal (464 kDa, pI 4.6) and SAP (30 kDa, pI 9), were used as models in this study to evaluate the protective ability of PAEs. It was surprising that L3 was able to achieve SAP mediated ribosome inactivation when SAP was strongly cationic at pH 4.5 during nanoparticle formation, although the electrostatic forces usually are regarded as inefficient for PAEs to bind to a cationic protein cargo. In contrast, PULsin and Pierce which are dedicated to the delivery of anionic peptides, antibodies and proteins,^51^ successfully delivered β-Gal with isoelectric point at 4.6 but failed to deliver cationic SAP. In addition, protein transfection using PULsin and Pierce requires serum free media to incubate protein complexes with cells, whereas PAEs developed here can be directly used with complete cell culture media and do not require changing media during the process, which simplifies the transfection procedure, and is promising for further in vivo applications. The optimal polymer to protein ratios were determined for both PAEs, 20 and 30 for L3PG and L3, respectively. Although the resulting larger particle size of these two ratios hinders the protein intracellular internalization, they are still the best conditions for both PAEs considering different aspects: (1) protein encapsulation capacity, (2) protein uptake level, (3) sufficient protein protection ability, and (4) targeted delivery ability of functional protein. To establish optimal RNP transfection conditions in HeLa-GFP cells, we performed all cytosolic protein delivery assays exclusively in HeLa cells. However, given the potential impact of cell type on protein delivery efficiency, it is imperative to evaluate PAEs-mediated protein transfection across various cell lines in future studies.

Lastly, we demonstrated that L3PG was capable of functionally delivering the CRISPR/Cas9 RNP for efficient gene editing. Compared to plasmid and message RNA (mRNA) encoding the CRISPR/Cas9 system, CRISPR/Cas9 RNP direct delivery has the advantage of avoiding the intracellular transcription and translation process, transient genome editing with reduced off-target effects, and high editing efficiency. However, application of CRISPR/Cas9 RNP based gene editing therapy has still been hindered by lack of ideal delivery methods. Herein, *in vitro* L3PG delivered CRISPR/Cas9 RNP resulted in 19% targeted gene editing in polyclone, which is comparable to the commercially available CRISPR/Cas9 RNP delivery reagent CMAX. It should be noted that L3PG delivery of CRISPR/Cas9 RNP contributed up to 3 times more targeted indels than with L3, which indicated PG modification on the PAEs not only improved its protein delivery capabilities, but also enhanced the stabilization of the CRISPR sgRNA cargo. This is a significant observation that can inspire further development of a delivery platform of CRISPR systems in the RNP format.

## Conclusion

In summary, we report here that the rationally designed guanidyl-rich PAEs demonstrate robust intracellular delivery capabilities and biocompatibility for functional cytosolic proteins with different isoelectric points and sizes. PG as end-capping on PAEs, enhanced the interaction between PAEs and cargos, and contributed to highly efficient protein delivery with undamaged bioactivities. This versatile protein delivery carrier can be a useful tool in biological research and various protein therapeutic applications. The superiority of PG modification in RNP delivery also provides a clue for further development of nonviral carriers in CRISRP RNP-based gene editing therapies.

## Materials and Methods

### Materials

For polymer synthesis, 1,4-butanediol diacrylate (BDA), 5-amino-1-pentanol (AP), 1,3-diaminopropane (DAP), *N*-(3-(dimethylamino)propyl)-*N*′-ethylcarbodiimide hydrochloride (EDAC), 4-(dimethylamino)-pyridine (DMAP), 4-guanidinobenzoic acid hydrochloride (GAH), deuterated chloroform (CDCl_3_), and lithium bromide (LiBr), were purchased from Merck Life Science (Darmstadt, Germany). Dimethyl sulfoxide (DMSO), dimethylformamide (DMF), acetone, methanol, and diethyl ether were ordered from Thermo Fisher Scientific (Waltham, MA, USA).

HeLa (a human cervical carcinoma cell line) was purchased from American Type Culture Collection (Manassas, VA, USA) and HeLa-GFP was purchased from GenTarget Inc. (San Diego, CA, USA). HEK-Gal8YFP cells were a gift from Prof. Duvall.^47^ Hank’s balanced salt solution (HBSS), 3 M sodium acetate, Dulbecco’s modified Eagle’s medium (DMEM) 6249, BSA, saporin from Saponaria officinalis seeds, and β-Gal were purchased from Merck Life Science. Fetal bovine serum (FBS), penicillin/streptomycin, Alamar Blue, goat antimouse IgG (H+L) highly cross-adsorbed secondary antibody, and Alexa Fluor 568, were ordered from Thermo Fisher Scientific (Dublin, Ireland). BSA-FITC, Gal Staining Kit, β-Gal Assay Kit, and LysoTracker Deep Red were ordered from Bio-Science Limited (Dublin, Ireland). β-Gal Galactosidase Detection Kit was ordered from Abcam (Cambridge, United Kingdom). Alt-R S.p. HiFi Cas9 Nuclease V3, CRISPR Cas9 sgRNA and Surveyor Mutation detection kit were purchased from Integrated DNA Technologies (Leuven, Belgium).

### PAEs Synthesis

To synthesize L3, 3.96 g of BDA monomer and 1.72 g of AP monomer were dissolved in DMSO (50% w/v), followed by bubbling with argon for 15 min at room temperature. The L3 was synthesized in a reaction at 90 °C until it reached the desired molecular weight, and the reaction was stopped by diluting with DMSO to 10% w/v. Afterward, 0.98 g of DAP was added into the mixture to end-cap the acrylate terminated base polymer by stirring at room temperature for 24 h. After completion of all reaction, L3 was precipitated by diethyl ether, further dried under a desktop vacuum, and stored at −20 °C.

The L3PG was further synthesized from L3. A 1.0 g portion of L3 was weighed and mixed with 0.23 g of EDAC, 0.15 g of DMAP, and 0.26 g of GAH, and all those monomers were dissolved in DMSO together to 10% (w/v). Then the reaction continued at room temperature and was stirring for 48 h, followed by 24 h of dialysis in methanol. Afterward, the PAEs-L3PG were precipitated using diethyl ether, dried under a desktop vacuum, and stored at −20 °C.

### PAEs Characterization (GPC and NMR)

The molecular weights of the polymers were determined by gel permeation chromatography (GPC) (Agilent Technologies, Ireland) equipped with a refractive index detector. Specifically, the polymer sample was diluted to ∼5 mg/mL with DMF, filtered through a 0.2 μm filter, and then injected into the GPC. The columns were eluted with DMF and 0.1% LiBr at a flow rate of 1 mL/min at 60 °C. Linear poly(methyl methacrylate) (PMMA) standards were used for calibration.

The chemical structure and composition of polymers were further confirmed with ^1^H NMR. Polymer samples were dissolved in CDCl_3_ and measured on a Varian Inova 400 MHz spectrometer (Edinburgh, United Kingdom).

### PAEs and Protein Nanoparticle Complexation

The polymer was dissolved in DMSO in 100 μg/μL stock solution and stored at −20 °C for the following studies. For polymer/protein complex preparation, each polymer and protein stock solution was dissolved in 25 mM sodium acetate (SA) to equal volume according to the desired polymer to protein weight ratios. Then, the polymer/SA solution was added to the protein/SA solution, mixed well by pipetting, and incubated for 15 min at room temperature to form polymer/protein complexes for subsequent studies.

### Protein/PAEs Complex Characterization

The sizes and zeta potential of PAEs/BSA complexes were measured using Zetasizer Pro (Malvern Panalytical, Worcestershire, United Kingdom) at 25 °C. The complexes were freshly prepared using the methods described above. The size of complexes was measured in a 10% (v/v) in DMEM media without FBS, and the zeta potential was determined in a 10% (v/v) H_2_O. Three individual tests were done for each sample.

The morphology of the polyplex was characterized by transmission electron microscopy (TEM). The PAEs/BSA complex was prepared as mentioned above. Then the complex solution was dropped onto 200 mesh copper grids and air-dried at room temperature for 20 min. The copper grids were further washed with distilled water three times to remove excessive salts. Images were captured on an FEI Tecnai T12 TEM system at a voltage of 120 V (Thermo Fisher Scientific).

### Protein Binding Assay

To test the protein binding ability, L3 and L3PG were complexed with BSA-FITC at W/W ratios from 5 to 80 and diluted using HBSS to 1.5 mL. The PAEs/BSA-FITC HBSS solution was centrifugated at maximum speed in a desktop centrifuge (Thermo Fisher Scientific), and 20 μL of supernatant was taken into a bottom-clear black 96-well-plate for fluorescence detection at *E*_x_/*E*_m_ = 488/525 in SpectraMax M3 multiplate reader (Molecular Devices, San Jose, CA, USA) immediately. Twenty μL of H_2_O was added back to the rest of the complex solution, mixed well, and incubated at 37 °C with shaking. At each sampling time point, the complex solution was centrifuged, and the supernatant was tested. The protein binding ratio was calculated using the following equation:
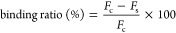
*F*_c_ represents the detected fluorescence intensity from the control samples that had the same amount of BSA-FITC without polymers. *F*_s_ represents the detected fluorescence intensity from the samples that were BSA-FITC complexed with L3 or L3PG. All of the conditions were measured by three individual repetitions.

### Cell Culture and Transfection

HeLa cells were cultured in DMEM 6429 with 10% FBS and 1% penicillin/streptomycin. HeLa-GFP and HEK-Gal8YFP cells were cultured in DMEM 6429 with 10% FBS, 1% penicillin/streptomycin, and 5% blasticidin, but in the transfection assays, both cell lines were growing in the full media without blasticidin. All the cells were incubated at 37 °C and 5% CO_2_ in a humidified incubator. HeLa and HeLa-GFP cells were seeded 24 h prior to transfection at cell density of 93 750 cells/cm^2^, while HEK-Gal8YFP cells were seeded at 31 250 cells/cm^2^ for transfection at ∼80% confluence. Polymer/protein complex was prepared freshly as described above, mixed with cell culture media, and then added to the cells.

### Cell Viability Assay

Cell viability was assessed 48 h post-transfection. Medium was removed, and cells were washed with 100 μL of HBSS per well. Then, 100 μL of Alamar Blue working solution (10% Alamar Blue in HBSS) was added to each well and incubated for 1 h under normal cell culture conditions as previously described. Absorbance was recorded at 570 and 600 nm by SpectraMax M3 multiplate reader. Untreated cells were used to normalize fluorescence values and were set as 100% viable. Wells containing only the Alamar Blue reagent were subtracted as background prior to obtaining the percentage of cell viability, calculated as follows:
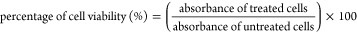


### Intracellular Delivery of BSA-FITC

At 24 h post-transfection, HeLa cells treated with PAEs/BSA-FITC were washed with 2% Trypan Blue for quenching the Alex fluorescein out of the cells, and then fluorescent images were taken by microscopy and flow cytometry was used to quantify the polymer/protein complex uptake.

### β-Gal Staining and Activity Assay

Cytostolic delivery of functional proteins such as β-Gal into HeLa cells was determined by intracellular β-Gal enzymatic activity measurements. In situ X-Gal staining kit and quantitative β-Gal enzyme activity assay kits were used according to the manufacture’s protocols. Briefly, 24 h post-transfection, HeLa cells were washed with phosphate-buffered saline (PBS) three times, fixed at room temperature for 10 min, and then washed with PBS twice following the incubation with X-gal substrate working solution at 37 °C for 2 h. The blue staining of the treated cells was checked under an epifluorescence microscope (Carl Zeiss, Dublin, Ireland).

β-Gal Assay Kit (Fluorometric) and β-Gal Galactosidase Detection Kit (ONPG) were used for quantifying β-Gal enzyme activity. In both assays, HeLa cells were lysed after incubation with PAEs/protein complex for 4 or 24 h in a bottom-clear, black 96-well-plate. Then, an β-Gal substrate (FDG or ONPG) working solution was added to the cell lysate. The samples tested with FDG were incubated at 37 °C for 2 h and then mixed with the stopping solution, and the fluorescence intensity was monitored at *E*_x_/*E*_m_ = 490/525 nm using SpectraMax M3 multiplate reader. Before adding the stopping solution to samples with ONPG, the mixture was incubated at 37 °C for 1 h and then the OD_420_ was detected by a SpectraMax M3 multiplate reader.

### Cytosolic Delivery of Saporin on HeLa Cells

To analyze the effect of saporin intracellular delivery, HeLa cells were treated with polymer/saporin in a similar way to other protein transfection. Polymer/BSA, unloaded polymer, free saporin, and transfection buffer were used as negative controls. Twenty-four hour (24 h) post-transfection, cell viability was tested as described in the cell viability assay.

### GFP Knockout by Delivery of Cas9 RNP

To evaluate the gene editing efficiency on HeLa-GFP cells, CRISPR sgRNA was mixed with Cas9 nuclease at designed molecular ratios and incubated at room temperature for 10 min to formulate the RNP *in vitro*. The RNP was further mixed with PAEs in a similar way to other protein as described above and added to the HeLa-GFP cells for transfection. Ninety six hours (96 h) post-transection, the treated HeLa-GFP cells were detached by 0.25% trypsin, and then washed with HBSS once. Subsequently, the collected cells were resuspended in 400 μL of HBSS for fluorescence analysis using CytoFLEX Flow Cytometer (Beckman Coulter Life Sciences, Indianapolis, IN, USA).

### Indels Detection Assay

96 h post-transfection, genomic DNA was isolated from cells using lysis buffer and precipitated by isopropanol and 75% ethanol. The targeted gene was amplified with the specific primers listed in Table S1 in the Supporting Information. Alt-R Genome editing mutation detection kit was used to detect the indels of the PCR amplicons following manufacturer’s instructions. The T7E1 digestion PCR products were separated on a 2% agarose gel and visualized in an iBright CL750 Imaging System (Thermo Fisher Scientific). The indels were also sequenced using Sanger sequencing by Eurofins Scientific (Luxembourg), and the Sanger sequencing data was analyzed using Tracking of Indels by Decomposition (TIDE) online tool (http://shinyapps.datacurators.nl/tide/).

### Investigation of Internalization Mechanism

PAEs/BSA-FITC transfection on HeLa cells was used to study the effect of PG groups on internalization mechanism. HeLa cells were preincubated at 4 °C, or preincubated in media with endocytic inhibitors including chlorpromazine (10 μg/mL), genistein (200 μM), and wortmannin (100 nM) for 30 min under normal cell culture conditions, followed by the addition of PAEs/BSA-FITC complex for transfection. The cell uptake was determined using flow cytometry after 4 h incubation with the complexes.

### Endo/lysosomal Escape Investigation

HeLa cells seeded on the cover slides were transfected by PAEs/BSA-FITC for 2 and 4 h. The treated cells were washed by 2% Trypan Blue in HBSS three times and followed by washing another three times using HBSS, then incubated with 50 nM of Lysotracker Deep Red and NucBlue (2 drop per mL medium) for 1 h at 37 °C. Before fixing cells with 4% formaldehyde solution in HBSS for 15 min at room temperature, the cells were washed with HBSS to remove remaining media. The cells were mounted using ProLong Glass Antifade mounting media and observed with a confocal laser scanning microscope (Carl Zeiss). The colocalization ratios between Lysotracker Deep red-stained endo/lysosomes and FITC-BSA were calculated using ImageJ Fiji software.^48^

HEK-Gal8YFP cells were transfected with PAEs/BSA for endo/lysosomal disruption investigation. After incubation with PAEs/BSA complexes for 2 and 4 h, the cells were washed by HBSS three times and stained using NucBlue (2 drop per mL media) at 37 °C for 30 min. The samples then were fixed in the same method as above, with HeLa cells, and mounted with the ProLong Glass Antifade medium for microscopy observation. For each condition, three slides were prepared as individual repetition, and six images were randomly taken from each slide for image analysis using ImageJ Fiji software. The Gal8-YFP images were processed according to the method described by Kilchrist et al.^47^ Cell numbers were determined via particle counting in the nuclear stain channel. The disrupted endosome was identified by adjusting the threshold of the YFP channel and semiquantified based on positive pixel counts and mean fluorescent intensity measurements.

### Statistical Analysis

All data are represented with the mean ± standard deviation (±SD), normally performing a minimum of three independent experiments. Analysis was carried out by one-way analysis of variance (ANOVA) with Dunnett’s multiple comparison or Tukey’s multiple comparison test, through the software GraphPad Prism 8.0 (San Diego, CA, USA). *p* values of <0.05 were considered significant ((*) *p* < 0.05, (**) *p* < 0.01, (***) *p* < 0.001, (****) *p* < 0.0001). Statistical significance was reported in the figure legends.

## Supporting Information Available

^1^H NMR spectrum of PAEs; TEM images of PAEs/BSA; PAEs/BSA-FITC cellular uptake results; PAEs/SAP transfection results; confocal images of HeLa cells; cell viability of treated HeLa-GFP cells; bioactivity of β-Gal released from PAEs/β-Gal complexes; fluorescence microscope images of HEK-Gal8YFP cells; GFP integrated density of electroporated HeLa-GFP cells; flow cytometry plots of HeLa-GFP and HeLa cells; PCR primer sequence; CRISPR sgRNA sequence; Indels on edited HeLa-GFP cell DNA (PDF)
