# Supplementary material for: Guanidyl-Rich Poly(β Amino Ester)s for Universal Functional Cytosolic Protein Delivery and Clustered Regularly Interspaced Short Palindromic Repeats (CRISPR) Cas9 Ribonucleoprotein Based Gene Editing
Source: ACS Nano. 2023 Sep 5;17(18):17799–810. doi: 10.1021/acsnano.3c03269 (PMC10540258; doi:10.1021/acsnano.3c03269)
Supplement: Supplementary file 1 — nn3c03269_si_001.pdf [file nn3c03269_si_001.pdf]

Guanidyl-Rich Poly (β Amino Ester)s for Universal Functional Cytosolic Protein Delivery and Clustered Regularly Interspaced Short Palindromic Repeats (CRISPR) Cas9 Ribonucleoprotein Based Gene Editing

Xianqing Wang[1,2,†]; Yinghao Li[1,†]; Xi Wang[2,]; Dario M. Sandoval[1]; Zhonglei He[1,2]; Sigen A[1,2]; Irene Lara Sáez[1,*]; Wenxin Wang[1,2,*]*

[1] Charles Institute of Dermatology, School of Medicine, University College Dublin, D04V1W8, Dublin, Ireland

xianqing.wang@ucdconnect.ie; yinghao.li@ucdconncet.ie; dario.sandoval@ucd.ie

[2] Research and Clinical Translation Center of Gene Medicine and Tissue Engineering, School of Public Health, Anhui University of Science and Technology, Huainan, 232001, China

zhonglei.he@brancabunus.com; sigen.a@aust.edu.cn

*Correspondence: xi.wang@brancabunus.com; irene.lara-saez@ucd.ie; wenxin.wang@ucd.ie

† Equal contributors.

Figure S1 ^{1}H NMR spectrum of L3 (400 MHz, CDCl3)

Figure S2 ^{1}H NMR spectrum of L3PG (400 MHz, CDCl3)

Figure S3 Transmission electron microscopy images of L3PG/BSA and L3/BSA.

Figure S4 Flowcytometry results of BSA-FITC cellular uptake at various polymer to protein weight ratios.

Figure S5 Saporin intracellular delivery mediated by L3 and L3PG.

Figure S6 Confocal images of HeLa Cells treated with PAE/BSA-FITC for 4 hours.

Figure S7 Cell viability of HeLa-GFP cells post-48 hours transfection with L3/RNP, L3PG/RNP and CMAX/RNP.

Figure S8 Bioactivity of β-Gal released from PAEs/β-Gal complexes.

Figure S9 Fluorescence microscope images of HEK-Gal8YFP cells for 2-hour treatment.

Figure S10 GFP integrated density of electroporated HeLa-GFP cells.

Figure S11 Flow cytometry plots of HeLa-GFP singlets.

Figure S12 Flow cytometry plots of HeLa singlets transfected by L3PG/BSA-FITC.

Figure S13 Flow cytometry plots of HeLa singlets transfected by L3/BSA-FITC.

Table S1 Primers for targeted DNA amplification.

Table S2 Sequence of sgRNA for GFP knock out on HeLa-GFP cells.

Table S3 Indels on target site of HeLa-GFP cell DNA.

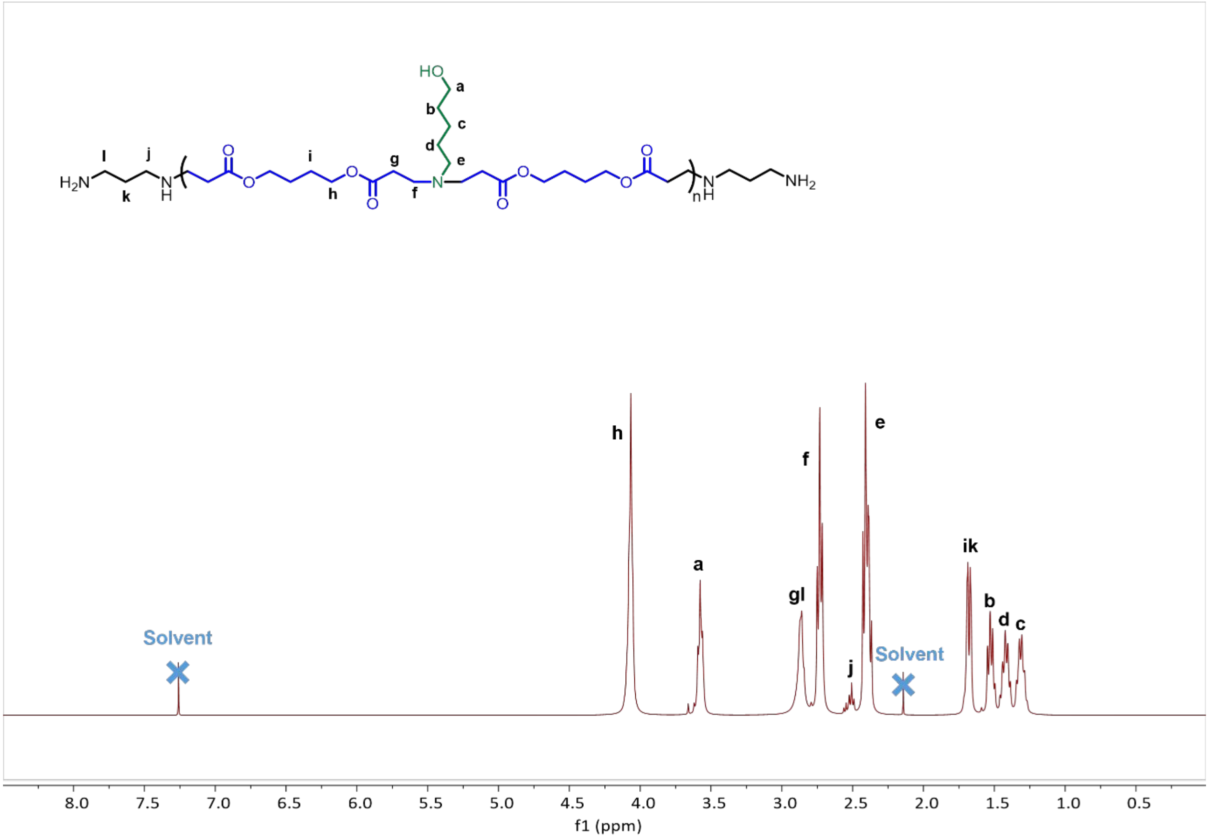

Figure S1 ^{1}H NMR spectrum of L3 (400 MHz, $CDCl_3$)

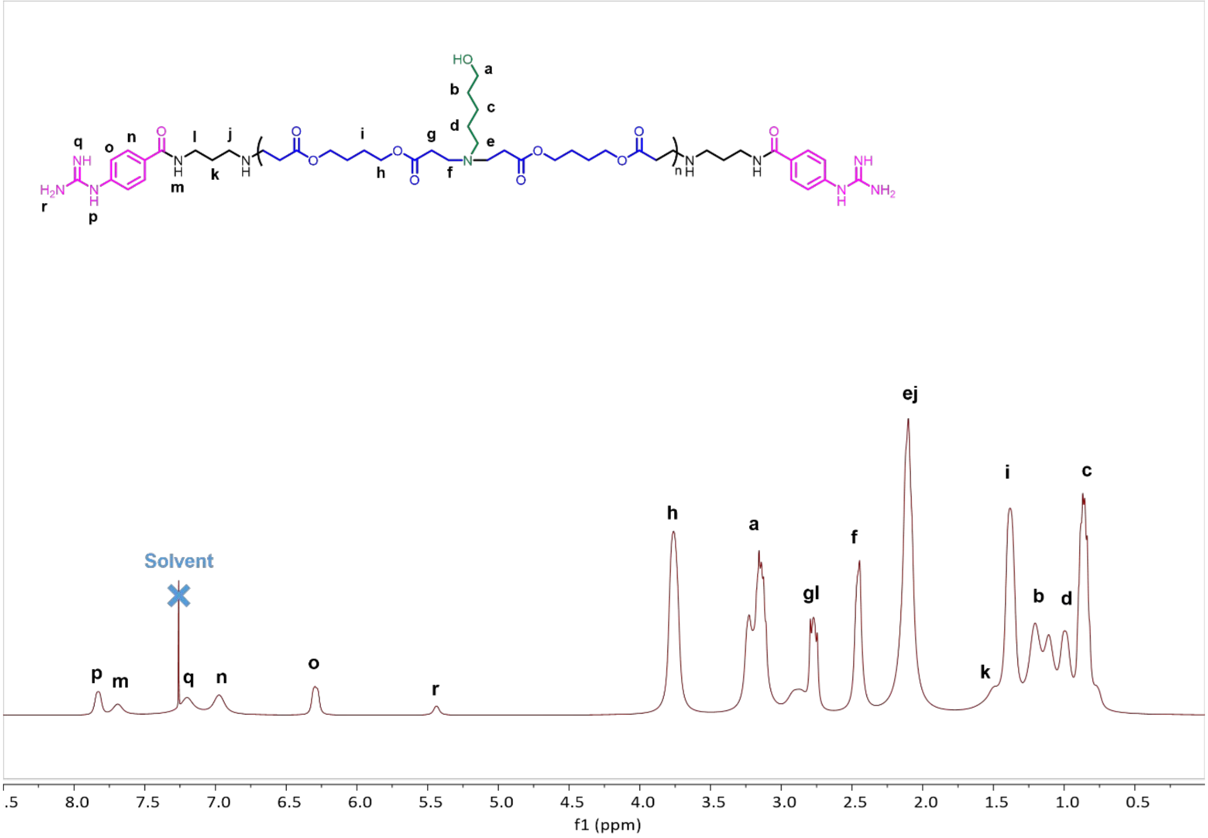

Figure S2 ^{1}H NMR spectrum of L3PG (400 MHz, $CDCl_3$)

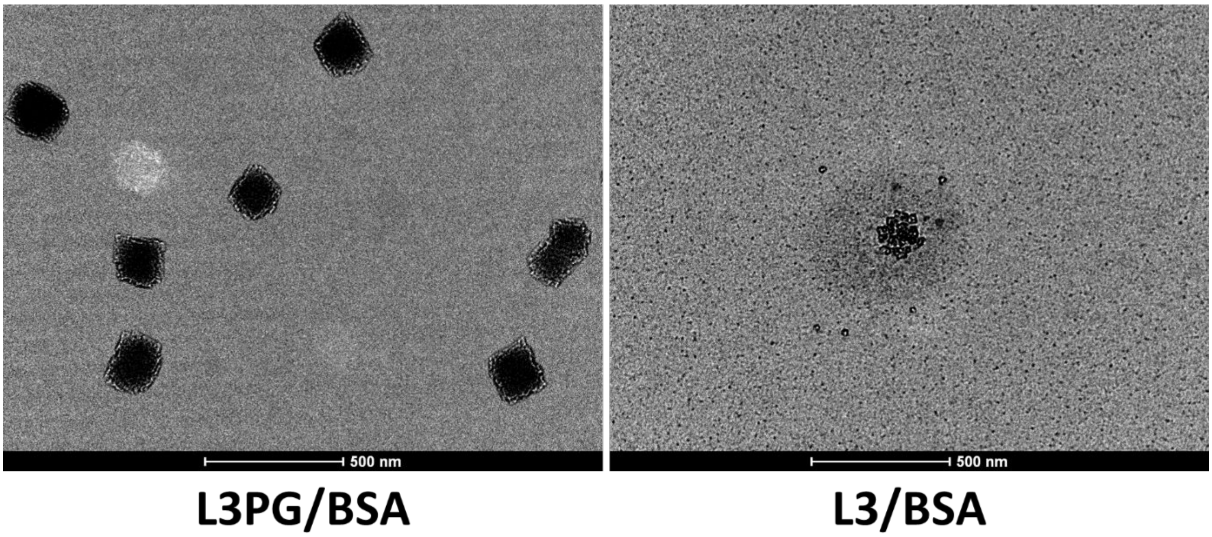

Figure S3 Transmission electron microscopy images of L3PG/BSA and L3/BSA. Scale bar =500 nm. W/W of 20 and 30 was used for L3PG and L3, respectively.

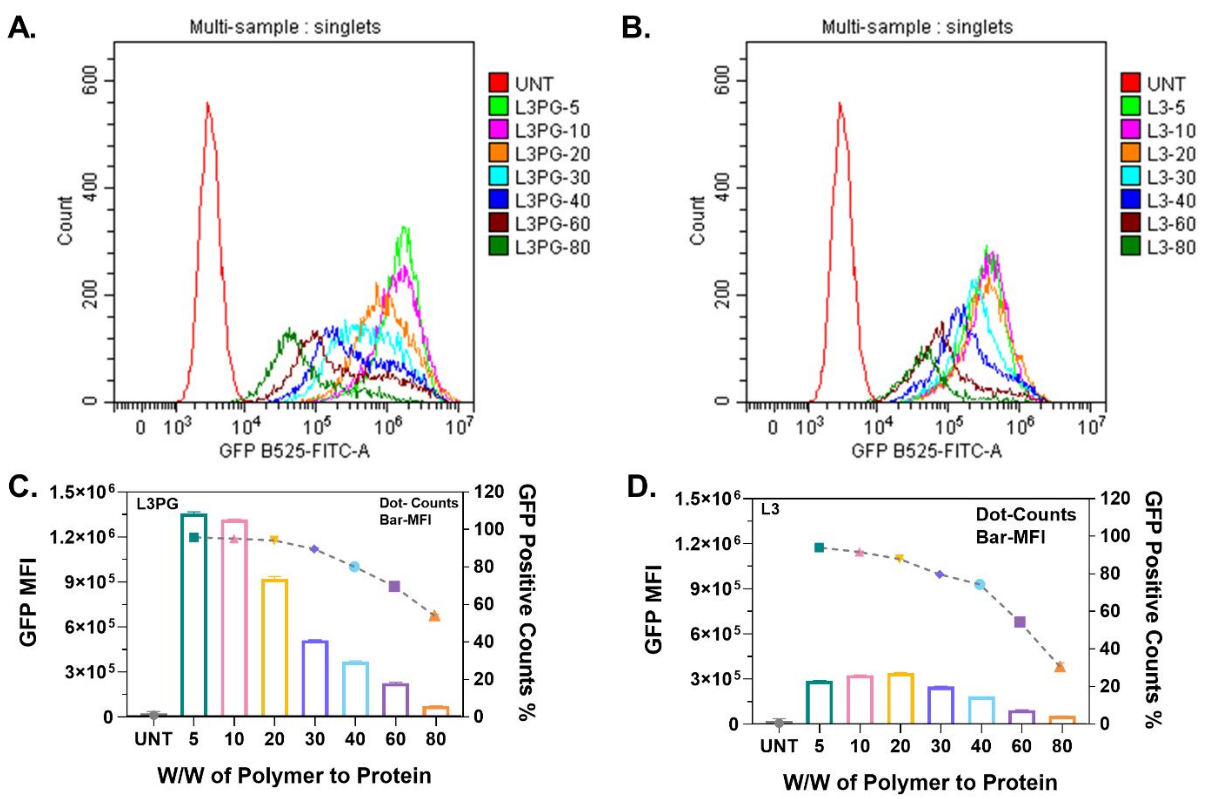

Figure S4 Flowcytometry results of BSA-FITC cellular uptake at various polymer to protein weight ratios. (A) GFP spectrum of HeLa cells treated by L3PG/BSA-FITC at w/w ranging from 5 to 80. (B) GFP spectrum of HeLa cells treated by L3/ BSA-FITC at w/w ranging from 5 to 80. (C) GFP mean fluorescence intensity of HeLa cells treated by L3PG/ BSA-FITC at w/w ranging from 5 to 80. (D) GFP mean fluorescence intensity of HeLa cells treated by L3/ BSA-FITC at w/w ranging from 5 to 80. Data was collected from 3 individual replicates of cells transfected after 24 hours.

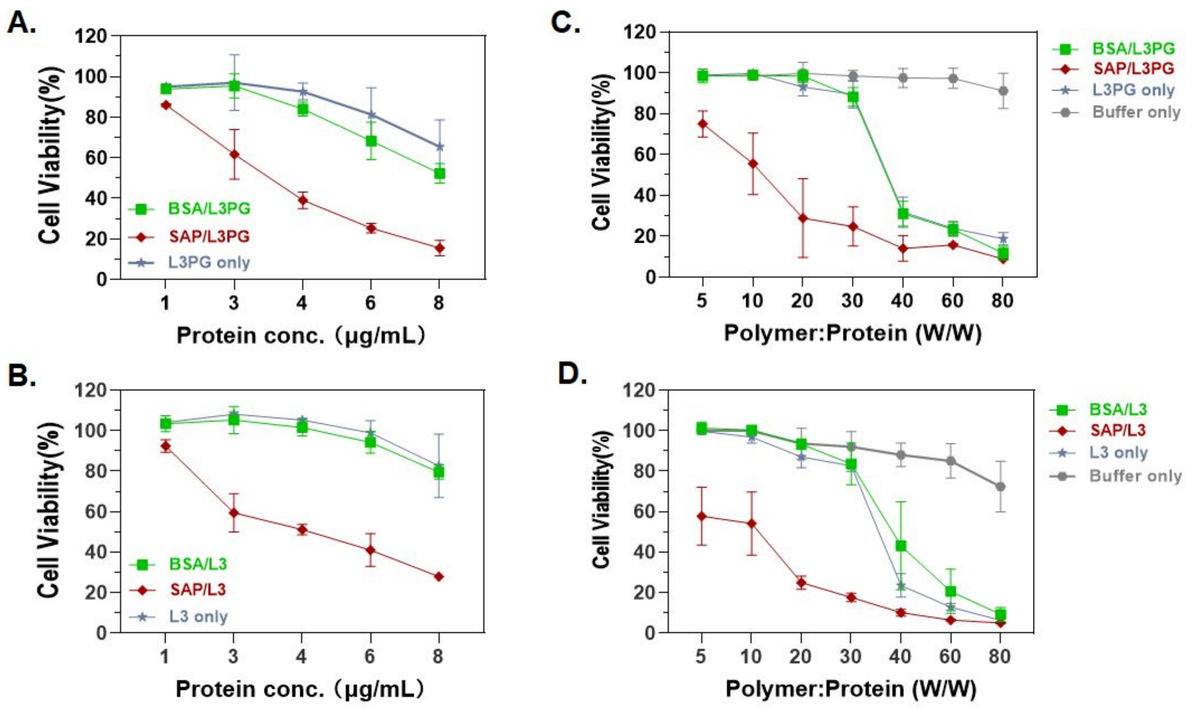

Figure S5 Saporin intracellular delivery mediated by L3 and L3PG. (A) Cell viability of Hela cells treated by SAP/L3PG, BSA/L3PG, and L3PG only at protein concentration ranging from 1 μg/ml to 8 μg/ml. W/W=30. (B) Cell viability of Hela cells treated by SAP/L3, BSA/L3, and L3 only at protein concentration ranging from 1 μg/ml to 8 μg/ml. W/W=30. (C) Cell viability of Hela cells treated by SAP/L3PG, BSA/L3PG, L3PG only and buffer only at protein concentration of 4 μg/ml, W/W ranging from 5 to 80. (D) Cell viability of Hela cells treated by SAP/L3, BSA/L3, L3 only and buffer only at protein concentration of 4 μg/ml, W/W ranging from 5 to 80. Data was obtained by flowcytometry. Data represented as mean ± SD (n=3).

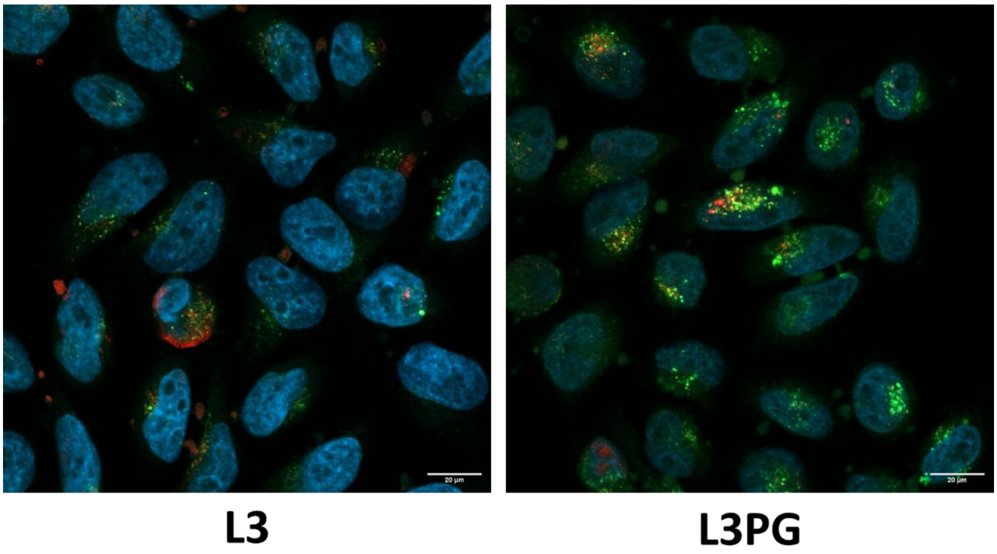

Figure S6 Confocal images of HeLa Cells treated with PAE/BSA-FITC for 4 hours. Green indicated BSA-FITC, red indicated endo/lysosomes, and blue indicated nucleus. Scale bar=20 μm. W/W of 20 and 30 was used for L3PG and L3, respectively.

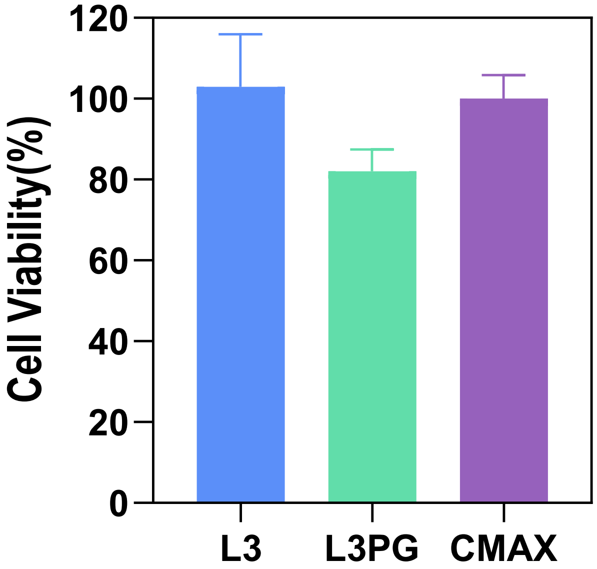

Figure S7 Cell viability of HeLa-GFP cells post-48 hours transfection with L3/RNP, L3PG/RNP and CMAX/RNP. W/W of 20 and 30 was used for L3PG and L3, respectively.

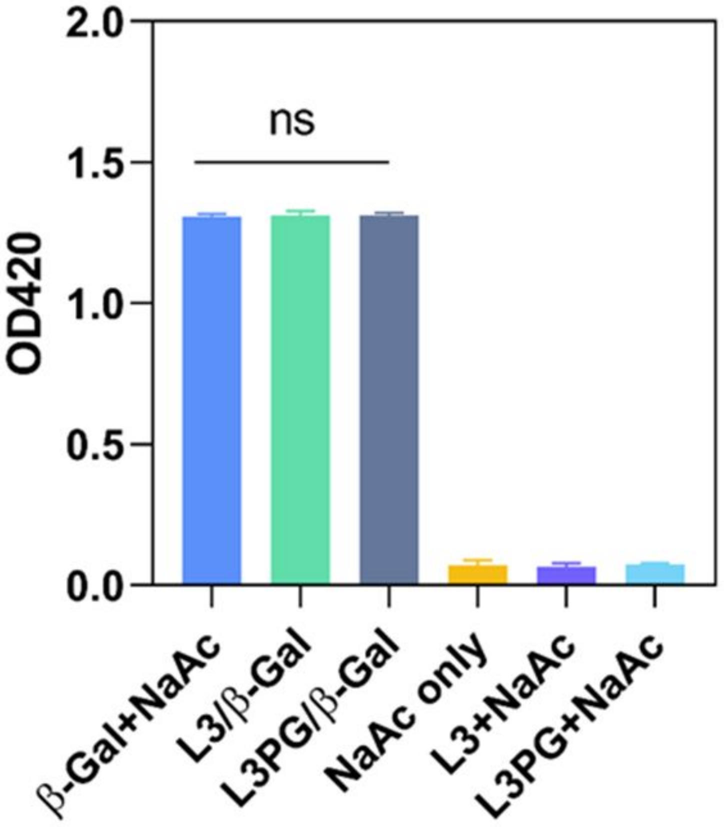

Figure S8 Bioactivity of β-Gal released from PAEs/β-Gal complexes. Bioactivity of β-Gal was tested using β-Gal Galactosidase Detection Kit (ONPG), when the PAEs/β-Gal complexes were incubated at 37˚C for 24 hrs. β-Gal in NaAc was used as positive control. ns, not significant.

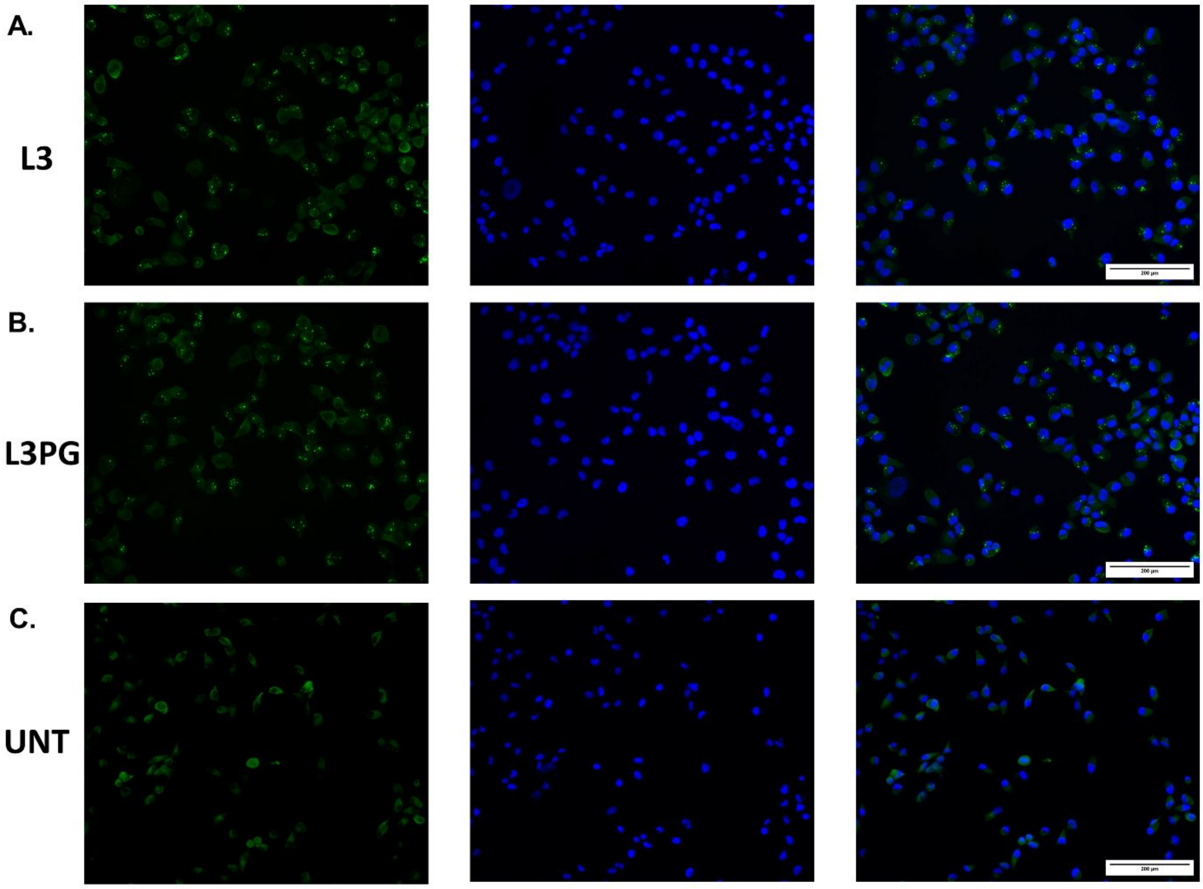

Figure S9 Fluorescence microscope images of HEK-Gal8YFP cells for 2-hour treatment. (A) HEK-Gal8YFP cells treated by L3/BSA. (B) HEK-Gal8YFP cells treated by L3PG/BSA. (C) Untreated HEK-Gal8YFP cells. Nuclei were shown in blue, the disrupted endosomes were indicated in green. W/W of 20 and 30 was used for L3PG and L3, respectively.

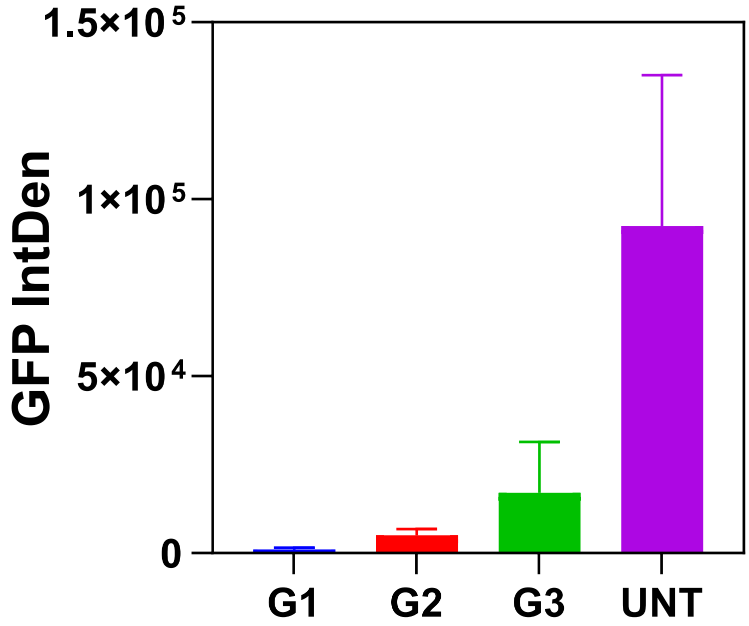

Figure S10 GFP integrated density of electroporated HeLa-GFP cells. Cells were electroporated with G1 (Cas9/GFP KO g1), G2 (Cas9/GFP KO g2), G3 (Cas9/GFP KO g3), and untreated HeLa-GFP cells.

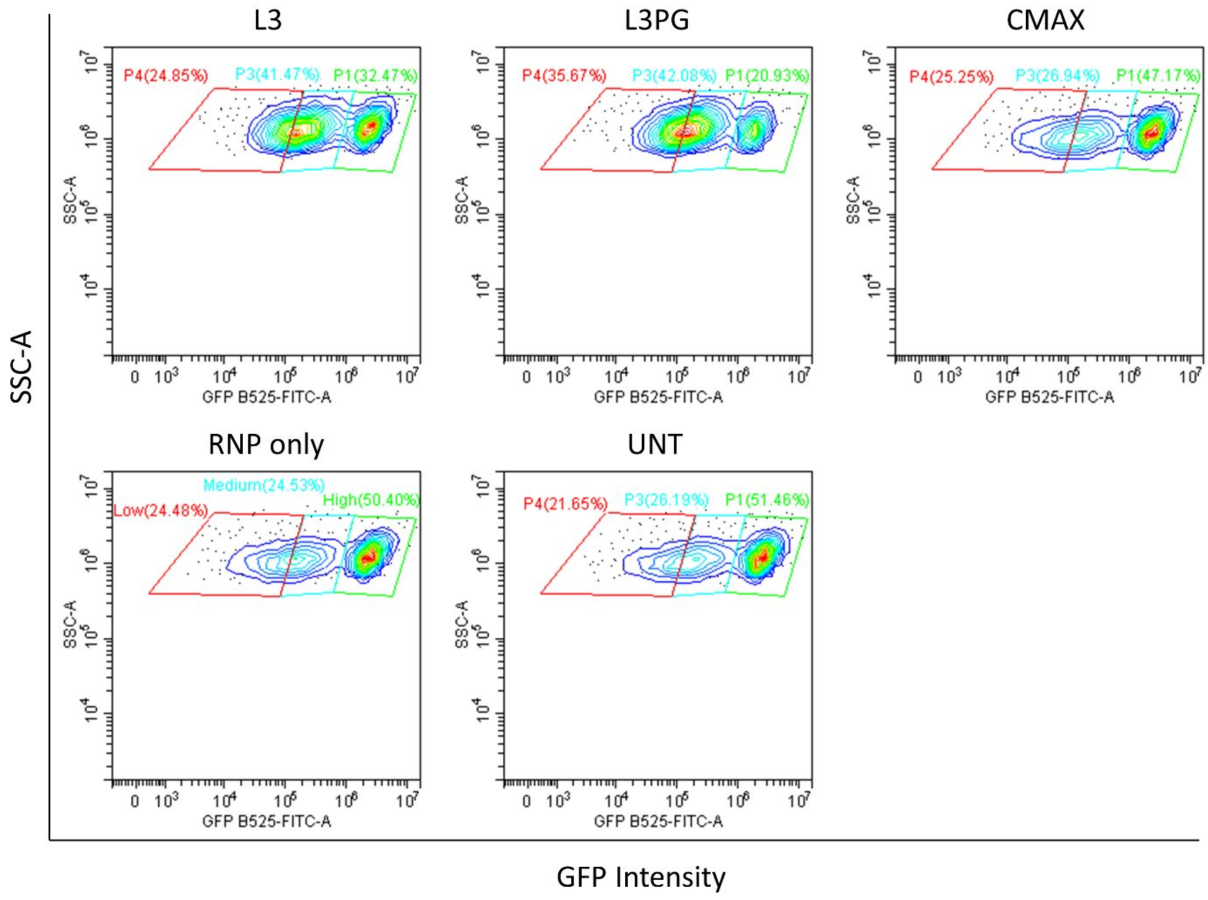

Figure S11 Flow cytometry plots of HeLa-GFP singlets. The HeLa-GFP cells were gated into three populations with high (P1, green), medium (P3, blue), and low (P4, red) GFP expression. W/W of 20 and 30 was used for L3PG and L3, respectively.

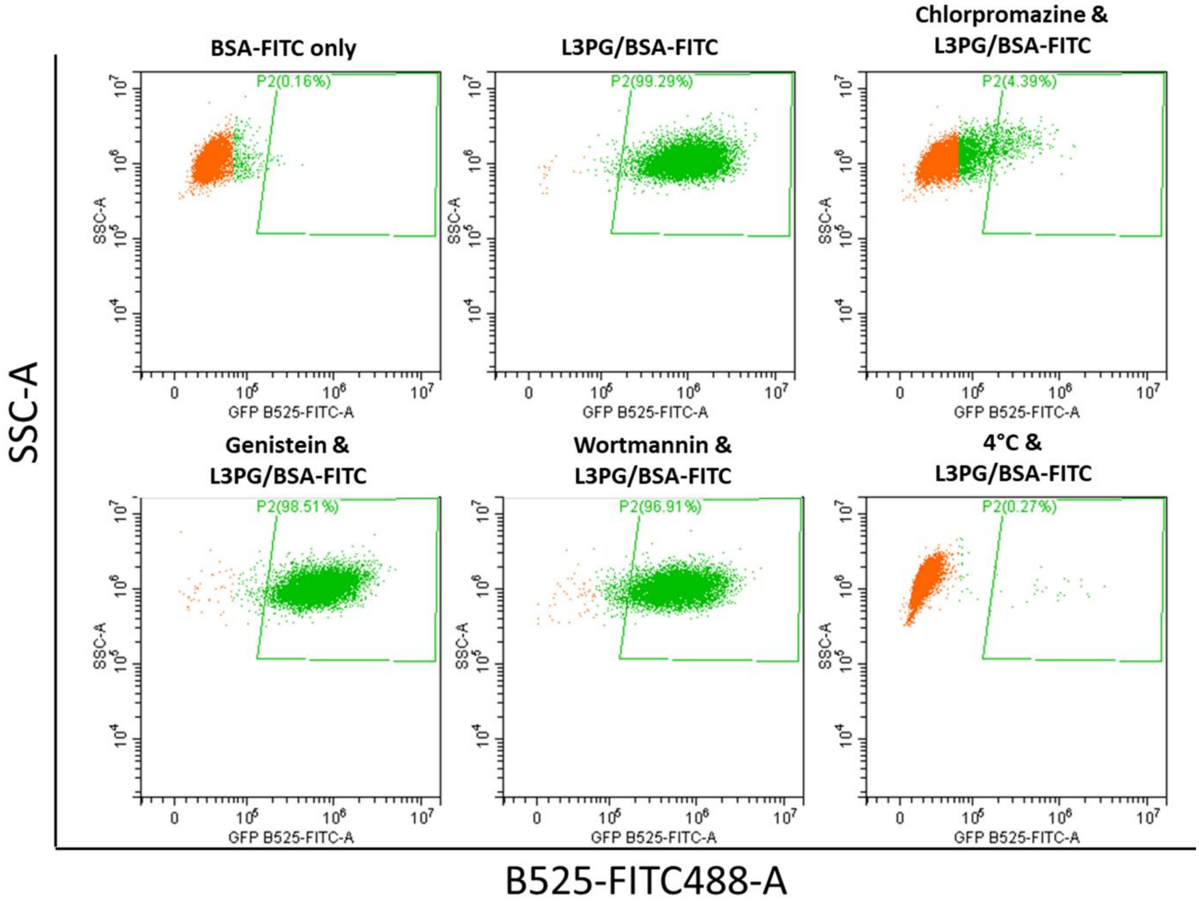

Figure S12 Flow cytometry plots of HeLa singlets transfected by L3PG/BSA-FITC. HeLa cells pre-treated with endocytosis inhibitors and transfected by L3PG/BSA-FITC at W/W of 20.

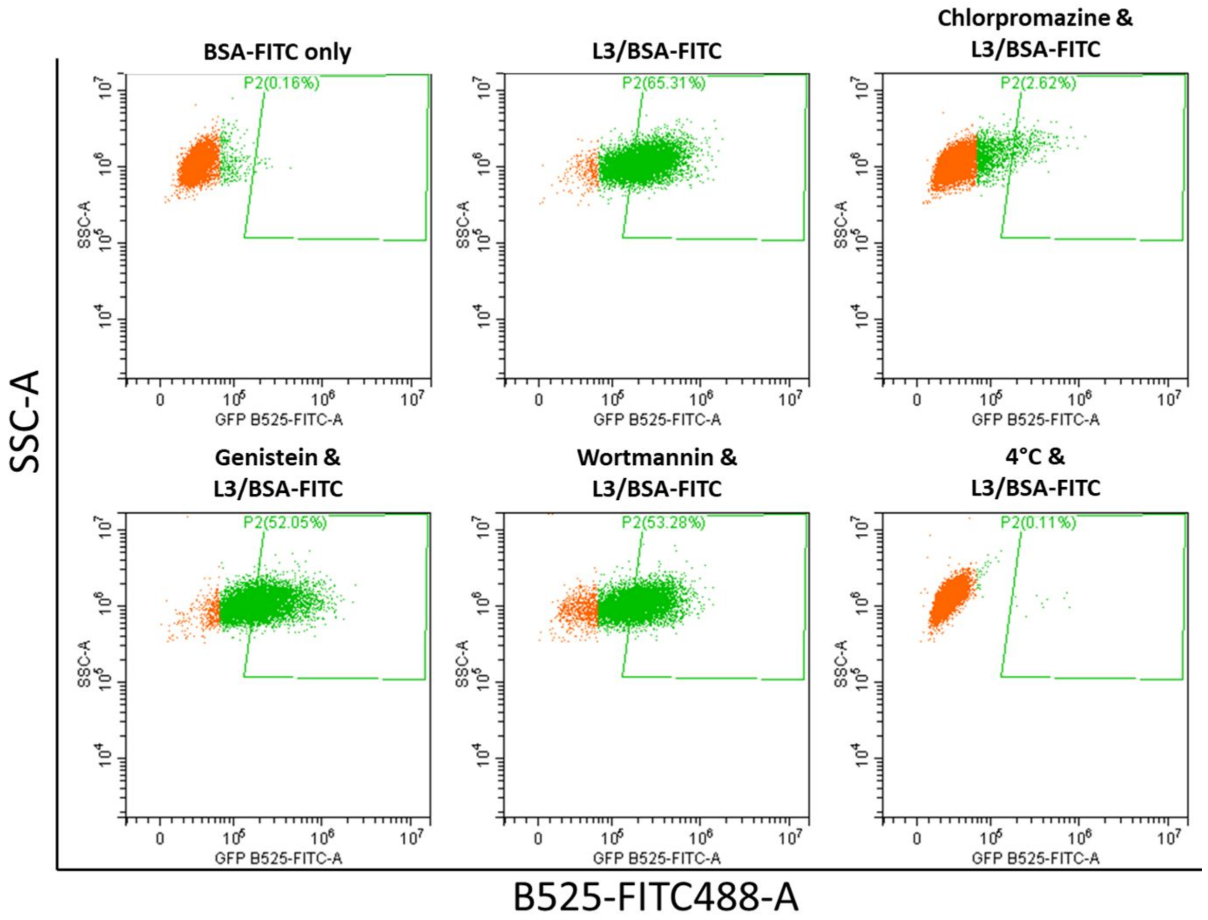

Figure S13 Flow cytometry plots of HeLa singlets transfected by L3/BSA-FITC. HeLa cells pre-treated with endocytosis inhibitors and transfected by L3/BSA-FITC at W/W of 30.

Table S1 Primers for targeted DNA amplification.

**Primers for PCR**	**Sequence**
Forward Primer	GCGACGTAAACGGCCACAAGT
Reverse Primer	TCAGCTCGATGCGGTTCACCAG

Table S2 Sequence of sgRNA for GFP knock out on HeLa-GFP cells.

**CRISPR Cas9 guide RNA**	**Sequence**	**Protospacer adjacent motif**
GFP K/O-1	CGCGCCGAGGTGAAGTTCGA	GGG
GFP K/O-2	TTCAAGTCCGCCATGCCCGA	AGG
GFP K/O-3	GAAGTTCGAGGGCGACACCC	TGG

Table S3 Indels on target site of HeLa-GFP cell DNA

**Treatment**	**Indels%**
Electroporation + GFP K/O g1	79%
Electroporation + GFP K/O g2	13%
Electroporation + GFP K/O g3	2%
L3PG + RNP	19%
L3 +RNP	6%

CMAX + RNP	20%
